# Three toxic gases meet in the mitochondria

**DOI:** 10.3389/fphys.2015.00210

**Published:** 2015-08-20

**Authors:** Richard A. Decréau, James P. Collman

**Affiliations:** ^1^Department of Chemistry (ICMUB Institute), University of Burgundy Franche-ComtéDijon, France; ^2^Department of Chemistry, Stanford UniversityStanford, CA, USA

**Keywords:** cytochrome c oxidase, biomimetic functional model, mitochondria, blood platelets, reversible inhibition, mitochondrial respiration, anticoagulants, electrocatalytic oxygen reduction

## Abstract

The rationale of the study was two-fold: (i) develop a *functional* synthetic model of the Cytochrome c oxidase (CcO) active site, (ii) use it as a convenient tool to understand or predict the outcome of the reaction of CcO with ligands (physiologically relevant gases and other ligands). At physiological pH and potential, the model catalyzes the 4-electron reduction of oxygen. This model was immobilized on self-assembled-monolayer (SAM) modified electrode. During catalytic oxygen reduction, electron delivery through SAMs is rate limiting, similar to the situation in CcO. This model contains all three redox-active components in CcO's active site, which are required to minimize the production of partially-reduced-oxygen-species (PROS): *Fe*-heme (“heme a3”) in a myoglobin-like model fitted with a proximal imidazole ligand, and a distal tris-imidazole *Copper* (“Cu_B_”) complex, where one imidazole is cross-linked to a *phenol* (mimicking “Tyr244”). This functional CcO model demonstrates how CcO itself might tolerate the hormone NO (which diffuses through the mitochondria). It is proposed that Cu_B_ delivers superoxide to NO bound to Fe-heme forming peroxynitrite, then nitrate that diffuses away. Another toxic gas, H_2_S, has exceptional biological effects: at ~80 ppm, H_2_S induces a state similar to hibernation in mice, lowering the animal's temperature and slowing respiration. Using our functional CcO model, we have demonstrated that at the same concentration range H_2_S can reversibly inhibit catalytic oxygen reduction. Such a reversible catalytic process on the model was also demonstrated with an organic compound, tetrazole (TZ). Following studies showed that TZ reversibly inhibits respiration in isolated mitochondria, and induces deactivation of platelets, a mitochondria-rich key component of blood coagulation. Hence, this program is a rare example illustrating the use of a functional model to understand and predict physiologically important reactions at the active site of CcO.

## Introduction

**Figure 1:**
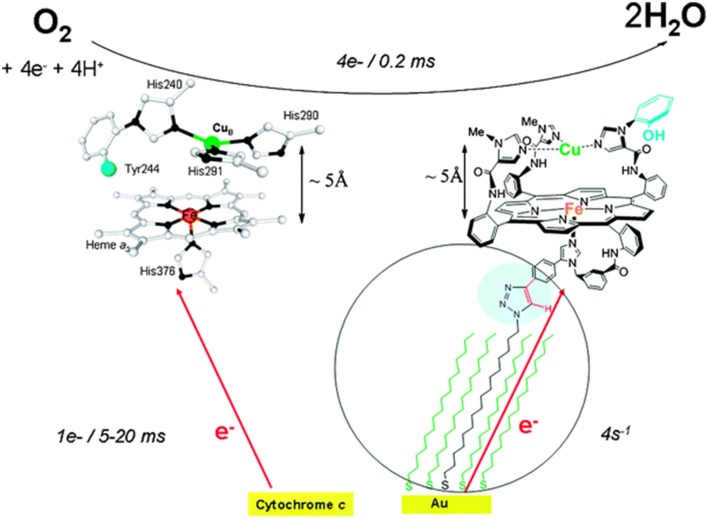
**Cytochrome c Oxidase heme ***a***_3_/CuB active site (left), and Model (right)**. Top: the 4e reduction of O_2_; bottom: biological electron transfer rates from cyt. C (left), and biomimetic rates from the electrode (right). Reproduced with permission from Decreau et al. (2010).

**Figure 2:**
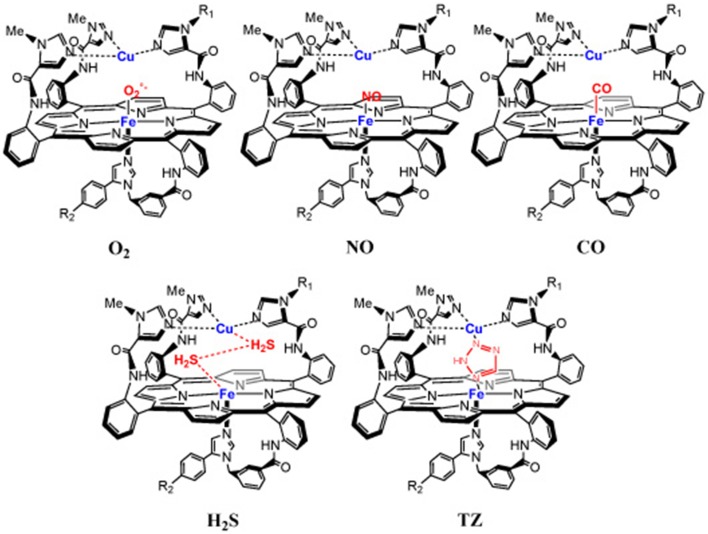
**Inhibition of a model of the CcO active site with gaseous- and non-gaseous ligands: NO, CO, H_2_S, TZ**.

Cytochrome c Oxidase (CcO) is a respiratory enzyme that achieves the 4e^−^ reduction of dioxygen to water, a process that is coupled to the formation of the body's energy currency, adenosine triphosphate (ATP). CcO is a transmembrane enzyme sitting in the mitochondria of eukaryotes, and it is also found in bacteria. This membrane-bound protein is composed of 13 subunits. X-ray diffraction studies show that it is composed of two hemes (cytochrome a and cytochrome a3), two copper centers (Cu_A_ and Cu_B_), and a tyrosine (Tyr_244_) (Figure 1). A four-electron pool is available in the CcO active site (heme *a3*/Cu_B_/Tyr_244_) to achieve dioxygen reduction during the final step of respiration. CcO is the last enzyme in the electron transport chain (i.e., complex IV), electrons are delivered from cyt. C one at a time at a very slow rate. The electrons subsequently tunnel from a bis-Cu_A_ (Babcock and Wikström, 1992; Iwata et al., 1995; Yoshikawa et al., 1998; Ludwig et al., 2001) and a six-coordinate heme a. This exergonic redox chemistry is used to generate a transmembrane proton concentration and electrostatic potential gradients. The biosynthesis of ATP is driven from such gradients, with proton pumped during reduction of O_2_, and translocations across the membrane. CcO reduces O_2_ all the way to water in a 4^e−^ process (Equation 1), without releasing partially reduced oxygen species (PROS), such as superoxyde (1^e−^ reduced), peroxyl (2^e−^ reduced), hydroxyl radical (3^e−^ reduced) being generated (Ferguson-Miller and Babcock, 1996). Such species are extremely toxic, they react with unsaturated fatty acids and induce cascade reactions. Hence, O_2_ reduction is a fundamental process which may lead to a variety of diseases either when it dysfunctions or when it is inhibited. Hence, we and others have tried to mimic the heme *a*_3_/Cu_B_ site, either through a synthetic or bioengineering approach (Liu et al., 2005; Kieber-Emmons et al., 2012; Miner et al., 2012). The goals pursued in our lab were two-fold: (i) mimic the active site from structural and functional standpoints (Figure 1), (ii) then examine our catalyst's reversible inhibition with three gaseous ligands that are encountered in the vicinity of CcO (Figure 2), and finally, to use this functional-model as a predictive tool for the inhibition of the actual CcO enzyme in respiring mitochondria with other non-gaseous ligands, and the deactivation of platelets (mitochondria-rich key components of blood coagulation). All data presented here (on the model, mitochondria, and platelets) are from publications in the literature.

(1) $$\begin{array}{l} {\text{O}}_{2}\;+8H^{+}\;4e^{-}\to 2H_{2}O+4H^{+} \end{array}$$

## Materials and methods

### Synthetic CcO model

#### Free model

Throughout the years, several versions of the CcO model were developped and fined-tuned (Collman et al., 1994, 1997a,b, 1998, 2002, 2004a) until the most accomplished version was obtained. The synthesis of heme protein model started with the ≪ picket fence porphyrin ≫ and other derivatives (such as capped/pocket porphyrin) that mimics the hemoglobin/myoglobin active sites (Collman et al., 1973, 1974, 1975a,b; Collman and Fu, 1999). The CcO model was synthesized in several convergent steps, developing porphyrin synthesis and face selection, imidazole synthesis, and metallations (Collman et al., 2004b,c; Decréau et al., 2007). This CcO model consists of an iron porphyrin (mimicking heme *a3*) fitted with a proximal imidazole (His_376_) and a distal Copper/ tris-imidazole pocket (mimicking Cu_B_, His_290_, His_291_, and His_240_, respectively) and also reproduces the FeCu distance (5 Å) found in the enzyme. A phenol is covalently bound to one imidazole mimicking the cross-link that occurs in His_240_-Tyr_244_ moieties. Throughout the synthetic sequence, all synthons were fully characterized, and this multiple step synthesis led to the final model with an 0.008% overall yield. Thorough characterization of the model utilized an array of spectroscopic and analytical techniques showing that the CcO model is isostructural with the CcO active site. The structure of the model's active site was confirmed by ^1^H-NMR (after reaction with CO to afford a low-spin diamagnetic state), and HRMS. Other biomimetic features, such as the FeCu distance, spin and oxidation states were carefully examined. Upon reaction of the oxidized Fe(III)Cu(II) model with N${}_{3}^{-}$ the resulting Fe(N_3_)Cu complex was found to be EPR silent, which suggested that azide bridges the two paramagnetic centers resulting in an antiferromagnetically coupling, which confirms that the FeCu distance is biomimetic (5 Å) (Collman et al., 2008a). Oxidation states of these models were confimed by ^1^H-NMR and EPR, and the characteristic UV/Vis absorption band [428/536 nm, i.e., 428 nm (Soret), 536 (Q bands)]. The spin state was also confirmed by the UV/Vis absorption band (either 428/536 in a six-coordinate (6C) ferrous model that contains a water cluster in the distal pocket, or 435/536 in the anhydrous model, i.e., a five-coordinate (5C) iron species) or using ^1^H-NMR with paramagnetic features in the anhydrous model. Moreover, the ν4 and ν8 spin state marker bands in the resonance Raman (*r*R) spectrum of the model show 2 bands corresponding to a mixture of high spin (HS) *S* = 2 (ν4: 1342 cm^−1^; ν8: 336 cm^−1^) and low spin (LS) *S* = 0 (ν4: 1356 cm^−1^; ν8: 380 cm^−1^) (Collman et al., 2009a,b). All models were stored under inert atmosphere before reaction with O_2_. Throughout the manuscript, the complete CcO model containing the two metals is depicted as an “FeCu model,” whereas a lower version that does not have the distal Cu_B_ is designated as “Fe-only model.”

#### Immobilized model

**Figure 3:**
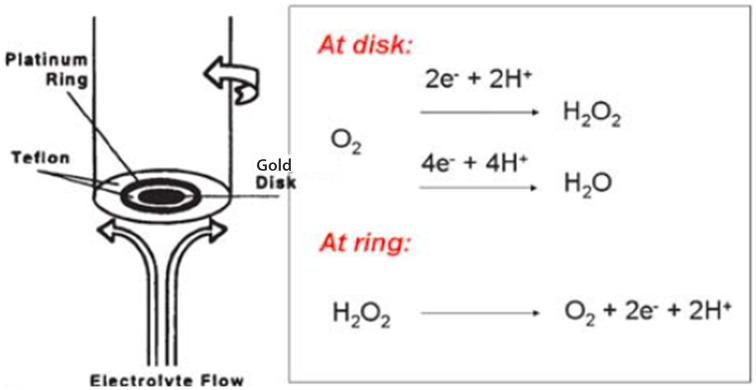
**Rotating ring disc electrode**. Reproduced with permission from Collman and Decréau (2008).

The alkyne-containing model was subsequently immobilized on liquid-crystalline azide-functionnalized self-assembled-monolayer (SAM)-coated-gold electrodes using click chemistry (Kolb et al., 2001; Tornøe et al., 2002). The length of the alkyl chains (i.e., in the linker that bridges the model to the electrode, and also the other chains (diluents) control the rate of electron delivery from the gold electrode to the model. These rates could be tuned to replicate the biomimetic electron-starving rates (i.e., *k*° = 4 s^−1^) by using C16/C18 alkyl chain links. Full characterization of the SAM electrode coverage was carefully achieved (goniometry, ellipsometry, blocking experiments) (Collman et al., 2004d, 2006a, 2009c; Devaraj et al., 2006; Collman and Decréau, 2008; Decreau et al., 2010). Such immobilization on SAMs enabled site-isolation of the CcO model, which is in stark contrast to adsorption on edge-plane graphite electrodes used with other systems (Collman et al., 2004a), where the electron delivery is several orders of magnitude faster than biological rates. Electrocatalytic studies were conducted using an assembly comprised of two electrodes, the rotating ring disc electrode system (Figure 3). In such an arrangement, the disc electrode where the model is immobilized is surrounded by a platinum ring electrode. Two currents are measured: (a) at the disc the current corresponding to O_2_-reduction by the model; (b) any ROS liberated during electrocatalytic reduction are swept hydrodynamically in the form of hydrogen peroxide from the disc to the outer platinum ring electrode where it is subsequently oxidized, affording the ring current. Examining both the ring current, and the catalytic current at the disc (by means of the Levich equation) (Levich, 1962), it is possible to measure the selectivity of the catalytic reduction of oxygen by the model catalyst. Alternatively, the model can be immobilized on inter-digitated array electrodes (IDAs) that exhibit about 60% collection efficiency, compared to 20% for RRDE (Collman et al., 2009c).

### Mitochondria

Mitochondria were isolated according to known protocols, either from fish liver or from yeasts. Fish liver mitochondria were prefered because of of their robust properties after isolation and because of their relative ease of procurement (Johnson and Lardy, 1967; Tan et al., 1996; Weinstein and Somero, 1998; Toninello et al., 2000). Fresh fish livers were dissected, minced, pulverized on a Dounce homogenezier and centrifuged (500 g). Upon filtration, a series of centrifugation/resuspension cycles (10,000 g at low temperature) was carried out. The final mitochondrial pellet was re-suspended in pH 7 buffer with a few additives, and the protein content was estimated by the Bradford assay (Bradford, 1976). Mitochondria were stored on ice and used within 12 h. Mitochondrial respiration studies were performed using a Clark electrode, prior to addition of ADP, malate and succinate in the respiration chamber. The inhibitor was added at 0°C and respiration rates measured 1 min later. Inhibition studies were performed at 20°C under stiring; reversibility measurements were performed upon washing in KH_2_PO_4_ buffer.

### Platelets

Blood specimen were obtained from volunteer donors following technical and ethics protocols developed in Loma Linda hospital. After phlebotomy, the blood-containing tubes were gently mixed by inversion (up to five times) and the blood was transferred to 5-mL plastic syringe. A detailed protocol previously developed to examine the time required for platelet clumping, sticking, and clotting (Pappas et al., 1994) was followed to examine the anti-coagulant properties of the selected heterocycles.

## Results

### Dioxygen

#### The ligand

Dioxygen is a neutral diatomic strong-field redox-active ligand. Ground state dioxygen is a triplet, with a unique electron configuration having two unpaired electrons in the π* molecular orbital. As previously discussed CcO reduces dioxygen with high selectivity, but when it malfunctions, toxic PROS are released.

#### Reactions with the model

##### Single-state turnover

**Figure 4:**
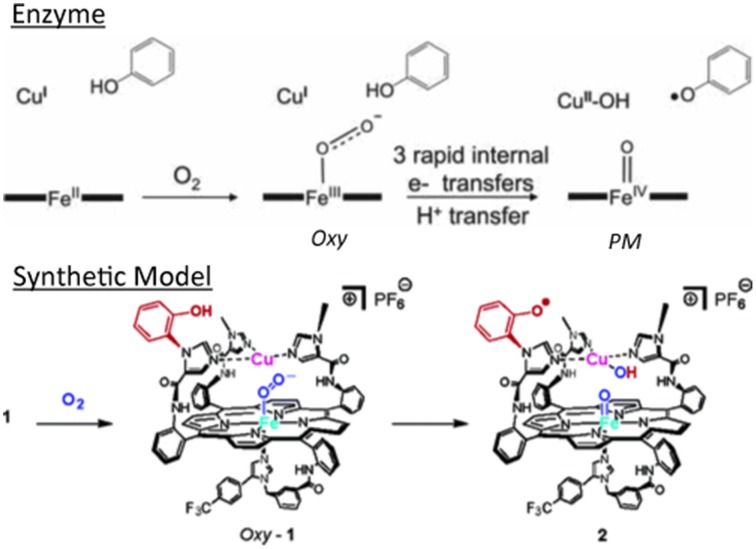
**Intermediates in the single-turn-over: enzyme (top) vs. model (bottom)**. Reproduced with permission from (Collman et al., 2007a,b).

The high-spin five-coordinate paramagnetic Fe(II)Cu(I) model was treated with O_2_. In this single turnover experiment, two intermediates were observed reminiscent of the intermediates found in the enzyme (Proshlyakov et al., 1998, 2000; MacMillan et al., 1999) (Figure 4).

##### Intermediate 1 (Oxy)

**Figure 5:**
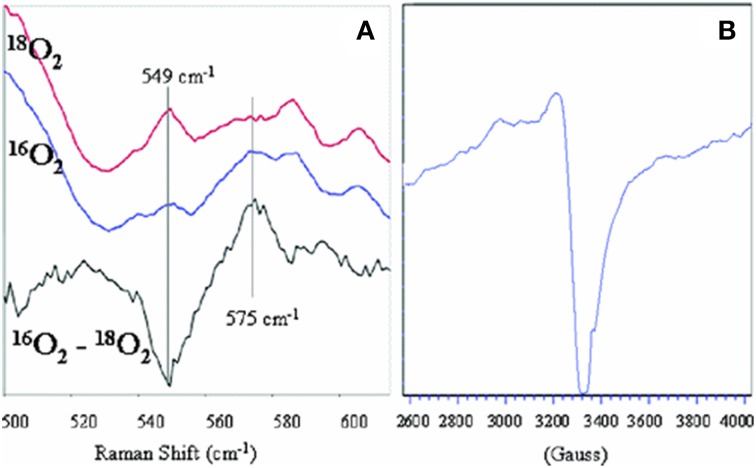
**Characterization of Oxygen Intermediates: resonance Raman spectrum of the dioxygen complex of 1 with ^18^O_2_, ^16^O_2_ [Oxy, (A), left], and the oxidation product showing the oxidation of Cu [PM, (B), right]**. Reproduced with permission from Collman et al. (2007a).

The reaction with dioxygen was examined with one metal site (the iron porphyrin) or two metal sites (the FeCu model). In order to bind dioxygen, a metal needs to be coordinatively unsaturated (so the ligand has a place to bind), and the metal needs to transfer an electron to dioxygen affording a coordinated ferric-superoxide species. Several bioinorganic features found in oxygen complexes of myoglobin/haemoglobin models were compared with that of the real enzymes, such as O-O and Fe-O_2_ stretching frequencies, Fe-O and O-O lengths, the Fe-O-O angle, spin and redox states, and the thermodynamic properties of O_2_ binding (Collman et al., 1973, 1974, 1975a,b, 1978, 2003; Collman, 1977; Momenteau and Reeds, 1994). The characterization of transition metal dioxygen complexes proposed by Vaska suggest an end-on superoxo in the case of iron-oxygen binding (Collman et al., 1976; Vaska, 1976). Recent L-edge XAS studies on oxy picket fence porphyrin (in solid state, under high vacuum, which leads to substantial loss of axial ligand) have attempted to examine the nature of the Fe-O_2_ bond in light of the Pauling (low spin *S* = 0), Weiss (low-spin *S* = 1/2) and McClure-Goddard-Harcourt Ozone (*S* = 1 ferrous) models (Wilson et al., 2013). In our FeCu model the first intermediate corresponds to an oxygen complex (421/550 nm), depicted as FeCuPhOH-O_2_. It is diamagnetic, it also has a characteristic oxygen isotope-sensitive resonance Raman band at 575/549 cm^−1^ (^16^O_2_/^18^O_2_) corresponding to the Fe-O stretch in an iron(III)-superoxide/Cu(I) species (Collman et al., 2003, 2007a) (Figure 5) reminiscent of Fe-^16^O_2_ stretches found in other natural heme-superoxide complexes such as Oxy-CcO (Varotsis et al., 1990). Other characteristic stretches of the iron-superoxo species were observed such as the ν4 band (spin state and redox state marker bands) at 1370 cm^−1^, which is typical of ferric-superoxo species (Burke et al., 1978; Walter et al., 1980; Varotsis et al., 1990). Moreover, the nature of the distal structure and the distal Cu_B_ were found to effect the ligand binding affinities and rates (for both O_2_ and CO) (Collman et al., 2009c).

##### Intermediate 2 (PM)

A second intermediate corresponds to an Fe(IV) = O/Cu(II)PhO° species that apparently results from O-O bond cleavage. It was characterized by EPR, reactivity (O atom transfer) and HRSMS (Collman et al., 2006b, 2007a). Such a ≪ PM ≫ intermediate is formed from the reaction of the *Oxy* intermediate with a phenol, either endogenous (in **1**), or exogenous (with simpler models that do not contain the built-in phenol moiety) to afford a hydroperoxyl species. These inter/intramolecular reactions lead to oxidation of the redox centers into Cu(II), oxoferryl and a phenoxyl radical.

##### Steady-state turnover

**Figure 6:**
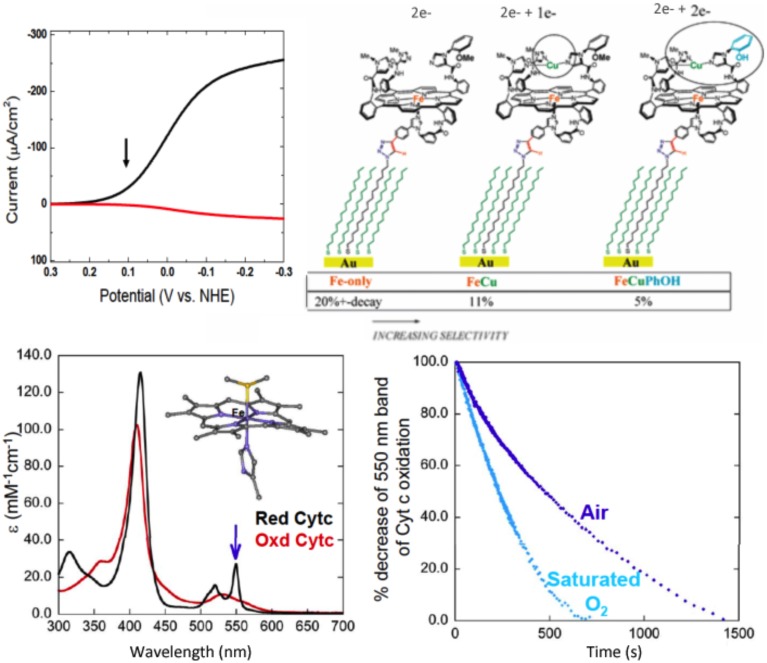
**Continuous reduction of O_2_**. **Top**: Model **1** is immobilized, the electron source is an electrode. Electrocatalytic O_2_ Reduction on slow SAM: Top left: Rotating ring-disk voltammograms of slow SAM modified with model 1 (arrow indicate at which potential PROS were measured). Top right: percentage of PROS detected on slow SAM modified with models bearing a 2e^−^, 3e^−^, and 4e^−^ pool, respectively. **Bottom**: model **1** is in solution, the electron source is cytochrome c. Bottom left: Kinetic traces showing a decrease of reduced cyt. C in the presence of 2% of **1** in aqueous buffer: acetonitrile mixture. Insert: absorption spectra of reduced and oxidized cyt c. Bottom right: reduced 1 (blue) and 1-iron only (red) in the presence of O_2_. Insert: absorption spectra of reduced-**1** (blue) and **1**-iron only (red). Reproduced with permission from Collman et al. (2007b); Collman et al. (2009b) and Decreau et al. (2010).

This model, fully equipped with a 4^e−^ reservoir (Fe, Cu, and phenol) and immobilized on slow SAM-electrodes (electron starving) catalyzed a continuous, selective O_2_ reduction yielding very little PROS (2%). This catalytic reduction proceeded continuously as it does in the enzyme (steady-state turnover). However, under such an electron-starved regime, the percentage of PROS goes from 2% with the FeCuPhOH (4^e−^ reservoir), but jumps to 11% with the FeCu model (3^e−^ reservoir), to more than 20% (and rapid decay) in the Fe-only system (2^e−^ reservoir), respectively (Collman et al., 2007b) (Figure 6).

A continuous catalytic reduction was also achieved by replacing the electrode as the source of electrons (when the model is immobilized on SAM) with the natural one-electron reductant cytochrome c [homogeneous reaction with the model in solution (2% loading)]. The latter reaction was studied spectrophotometrically (Figure 6). Monitoring the concentration of oxygen showed that 3.9 equiv. of Cyt. C are oxidized per molecule of O_2_ consummed. Such a result is consistent with a stochiometric four-electron oxidation. Oxygen binding was shown to be the rate-determining step (< 0.01 s^−1^) unlike electron transfer from Cyt. C to the oxidized Fe(III)Cu(II)PhOH model (1.2 s^−1^), or the O-O bond cleavage (Collman et al., 2009b).

### Nitric oxide

#### The ligand

Nitric Oxide (NO) is a critical regulator and messenger molecule employed to regulate physiological processes in mammals. NO is a stable but reactive free radical, where the unpaired electron resides in a π^*^ molecular orbital (McCleverty, 2004). NO is a monomeric diatomic and paramagnetic molecule that reacts with dioxygen. NO can be either oxidized into a nitrosonium NO^+^ species or reduced into the nitroside anion NO^−^, which are isoelectronic with CO (NO^+^) and O_2_ (NO^−^). NO is redox active in solution with a standard reduction potential of NO^+^ to NO estimated to be +1.2 V vs. NHE (Standbury, 1989), NO to ^3^NO^−^ (−0.8 V) and ^1^NO^−^ (−1.7 V). NO binds to transition metals, the nitrosyl complexes are described by the Enemark and Feltham formalism because of the difficulty of assigning formal oxidation states to both NO and the metal in these complexes (Enemark and Feltham, 1974; Westcott and Enemark, 1999). In mammals NO is produced by NO-synthase (NOS), one type is located near mitochondria (mtNOS) (Ghafourifar and Richter, 1997; Giulivi et al., 1998), producing a steady flux of NO in the vicinity of CcO. The [NO]/[O_2_] ratio in mitochondria is 0.001. NO binding to the CcO active site is reversible, but NO is a fierce competitor to O_2_, as it rapidly and strongly binds to CcO (k_on_ 10^8^ M^−1^s^−1^, *K*_D_ < 10^−9^ M; the ferrous heme strongly binds NO (*K*_*eq*_ = 10^9^) whereas the dioxygen affinity is much lower *K*_eq_ = 0.1) (Petersen, 1977; Stamler et al., 1992; Ford and Lorkovic, 2002; Brunori et al., 2006). NO is a competitive inhibitor of CcO (*K*_1_ = 0.27 μM). There is a conundrum; CcO should be permanently inhibited by NO in the mitochondria, but this does not occur.

#### Reaction with the model

##### Authentic NO-complexes

**Figure 7:**
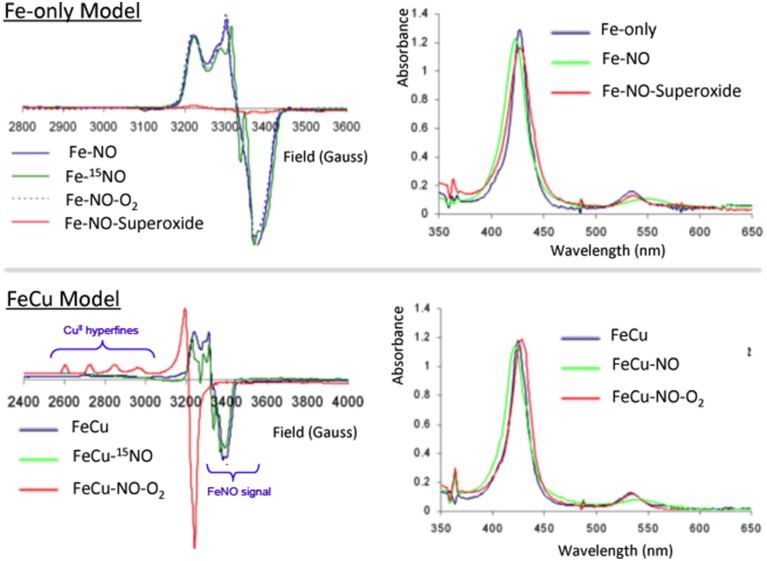
**Inhibition by NO: EPR and absorption spectra of the NO derivatives and their reaction with O_2_ and superoxide for Fe-only and FeCu model complexes**. Reproduced with permission from Collman et al. (2008b,c,d).

To examine the biomimetic character of the FeCu model with respect to this gaseous ligand, each individual metal site was first examined in a series of control reactions (Collman et al., 2008b,c,d). The iron-only species reacts with NO (1 equiv.) to afford a species depicted as Fe-NO, which is a stable six-coordinate low-spin iron nitrosyl species that was characterized by a series of spectroscopic tools, such as EPR (*S* = ½ gz 2.078, gy 2.015, gx 1.97, with multiple ^14^N superhyperfine in the Ay region with perturbation upon enrichment with ^15^NO isotope comparable with six-coordinate ferrous iron-nitrosyl with a proximal imidazole such as the ferrous nitrosyl species in CcO (Yonetani et al., 1972; Stevens et al., 1979; Yoshimura, 1991; Makino et al., 1999), HRMS, and UV/Vis (425/548 nm). Similarly, the FeCu species reacted with NO affording an adduct with spectroscopic features comparable to that observed with the iron-only case (UV/Vis, EPR, HRMS). The Fe-NO moieties were characterized by vibrational spectroscopy, revealing an isotopically sensitive band at 1630/1600 cm^−1^ (Figure 7) (Collman et al., 2008b,c,d) that may be compared to that of nitrosyl species, νNO^+^ = 2150 − 2400 cm^−1^, νNO = 1875 cm^−1^, νNO^−^ = 1470 cm^−1^) (Cheng and Richter-Addo, 2000). Also it manifests an isotopically sensitive resonance Raman band νFe-N_(NO)_ = 581/545 cm^−1^ compared with νFe-N_(Imidazole)_ = 281 cm^−1^ (Collman et al., 2008b,c,d).

##### The O_2_/Cu_B_ system reverts NO inhibition

However, a striking difference between these two iron-nitrosyl species (Fe-NO and FeCu-NO), is their reactivity toward dioxygen (Collman et al., 2008c) (Figure 7). The iron-only nitrosyl model (Fe-NO), does not react with oxygen. For convenience, the resulting iron nitrosyl model in contact with air is designated as Fe-NO-O_2_. No change in its EPR and UV/Vis absorption spectra were observed compared to that of Fe-NO. Hence, this demonstrates that Fe-NO is stable in the air. When Cu_B_ is present in the distal site, the Fe(NO)Cu species spontaneously reacts with dioxygen to afford an Fe(II)Cu(II) species: the UV/Vis spectrum (429/539 nm) is reminiscent of a ferrous species, whereas EPR indicates the appearance of a Cu^2+^ signal (type 2, *S* = 1/2, *g*// = 2.40, *g* = 2.082, *A*// = 122 cm^−1^) and the disappearance of the *S* = 1/2 Fe(NO) signal. Such a result indicates that the distal Cu may play a role: this question was examined in light of the stability of the Cu-O_2_ moiety that results from the intermolecular reaction between distal Cu(I) and O_2_, and the potential of Cu (0 mV) which makes subsequent reduction of O_2_ to O${}_{2}^{-}$ feasible (Vanbuuren et al., 1972). Such an intermediate species is known to dissociate to afford a Cu(II) complex and free superoxide. We suggested that a subsequent reaction of superoxide with the iron nitrosyl may occur, affording a ferrous heme and a putative peroxynitrite species that should disproportionate to form nitrate. Note that, under physiological conditions, peroxynitrite would be reduced to nitrite, instead (Koppenol et al., 1996). Such conclusions considering the superoxyde proposal is supported by a control reaction examining the reactivity of free superoxide with the air-stable iron-only nitrosyl Fe-NO complex. As for the FeCu-NO compound, an Fe(II) species forms (and the formation of the same nitrogen products is proposed), labeled Fe-NO-O${}_{2}^{{}^{\circ}-}$: this complex, which has characteristic ferrous heme absorption features (428/538 nm), has lost its iron nitrosyl *S* = 1/2 EPR signal. Moreover: EPR measurements at 4 K rule out the formation of a high-spin ferric complex. Overall, this set of experiments addressing the reaction of NO/O_2_ with a CcO model, suggests that inhibition of the CcO active site proceeds through the formation of a stable iron-nitrosyl species, and then the nitrosyl species is removed only in the presence of and through reaction with superoxide, which can only be formed in the presence of a distal Cu(I). The presence of Cu(I) and a constant supply of electrons result in protection of CcO against NO inhibition.

### Carbon monoxide and cyanide

#### The ligands

Beside nitric oxide, other ligands are well known to be inhibitors of CcO: carbon monoxide CO (*K*1 = 0.32 μM) and cyanide CN^−^ (Petersen, 1977). These are also strong-field π-acceptor ligands. These ligands form much stronger complexes with Fe(II) and Fe(III) compared with O_2_. In humans these poisonous ligands result from either metabolism of heme for CO (Otterbein et al., 2003), or cyanogenic glycosides (originating in fruit) for CN^−^ (Aregheore and Agunbiade, 1991). In CcO CN^−^ binds tightly to the oxidized active site (*Kd* = 1 μM) (Vanbuuren et al., 1972), which should inhibit enzyme turnover because it affects the reduction potential of Fe(III)/Cu(II) to Fe(II)/Cu(I) by the heme-a center located in the vicinity of the Fea_3_/Cu_B_/Tyr^244^ arena (Wainio and Greenlees, 1960; Vanbuuren et al., 1972; Kojima and Palmer, 1983).

#### Reactions with the model

##### CO/CN complexes

**Figure 8:**
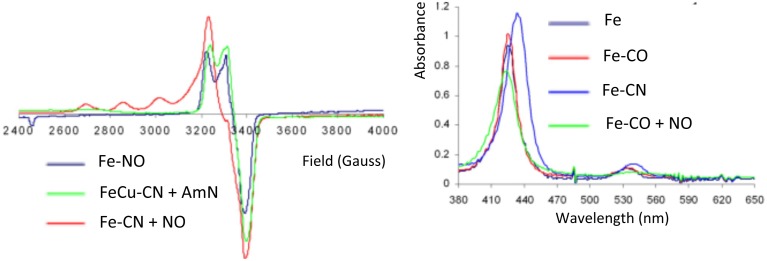
**Inhibition by CO/CN**. EPR and Absorption spectra of CO and CN derivatives of Fe-only and FeCu models, and their reaction with NO and AmN, respectively. Reproduced with permission from Collman et al. (2008c).

The oxidized Fe-only model (415/519 nm) reacts with CN^−^ to afford a species having characteristic UV/Vis bathochromic shifts consistent with a Fe-CN species (433/541 nm). Moreover, reaction of CO with the Fe-only complex affords a Fe-CO species (427/538 nm) (Collman et al., 2008c) (Figure 8). Previously, Fe-only and FeCu CO derivatives were shown to be stable in air and have been characterized by IR (νC-O 1950 cm^−1^ compared with 1959 cm^−1^ in CcO (Calhoun et al., 1993; Uno et al., 1994; Collman et al., 2002). It was therefore possible to characterize these low-spin, diamagnetic complexes (*S* = 0) by ^1^H-NMR.

##### O_2_/Cu_*B*_ and NO reverse cyanide (CN^−^) and carbon monoxide inhibition (CO)

##### Cyanide

In the scheme previously discussed, the reactivity with superoxide was examined, where superoxide accounts for Cu(I)-O_2_ that dissociates into Cu(II) and superoxide, which corresponds to the 1^e−^ reduction of O_2_. In the presence of superoxide, the ferric iron-only Fe-CN species is reduced to a CN-bound ferrous species labeled Fe-CN-O${}_{2}^{{}^{\circ}-}$ (428/538). In this ferrous complex CN is not as tightly bound as it is in the ferric case. Fe-CN reacts with NO (1 equiv.) affording Fe-CN-NO, that exhibits the characteristic UV/Vis and EPR features of an iron nitrosyl species (Figure 8); this result suggests that NO replaces CN^−^ in ferrous hemes (Collman et al., 2008c). Similarly, the reduced FeCu model reacts with CN^−^ affording a FeCu-CN species. This complex reacts with amyl nitrite (AmN) to afford a FeCu-CN-AmN derivative, the EPR signal of which exhibits a total spin integration (against a standard) that corresponds to two paramagnetic species, a four-line hyperfine feature suggests a Cu(II) species, and another feature at 3400 G is consistent with an *S* = 1/2 iron-nitrosyl species. This result is reminiscent of the inhibition described previously requiring a distal Cu: (i) reaction does not occur when Cu_B_ is not present in the distal cap, (ii) Cu_B_ is a 1^e−^ reductant of AmN that affords amyloxide, Cu(II), and NO subsequently replaces the CN^−^ ligand.

##### Carbon monoxide

The reaction of the ferrous Fe-only model with CO leads to a ferrous carbonyl (Fe-CO) species (Collman et al., 2002). The reaction of this Fe-CO complex with NO results in a complex depicted as Fe-CO-NO, the UV-Vis and EPR features of which correspond to that of Fe-NO (Collman et al., 2008c) (Figure 8). This result allowed us to conclude that NO displaces CO. Similarly, the FeCu-CO complex (fully reduced, i.e., ferrous/cuprous) reacts with AmN following the same mechanism described above, and leads to FeCu-CO-AmN, namely FeCuNO. The corresponding experiment performed with myoglobin shows the same UV-Vis features going from Mb-CO (423/544/582) to Mb-NO (424/551/582) (Bowen, 1949; Yonetani et al., 1972).

In the presence of NO, these complexes undergo replacement of a poisonous ligand affording diamagnetic iron-nitrosyl species (depicted as Fe-CN-NO and Fe-CO-NO, respectively) each having a characteristic *S* = 1/2 EPR signal.

**Figure 9:**
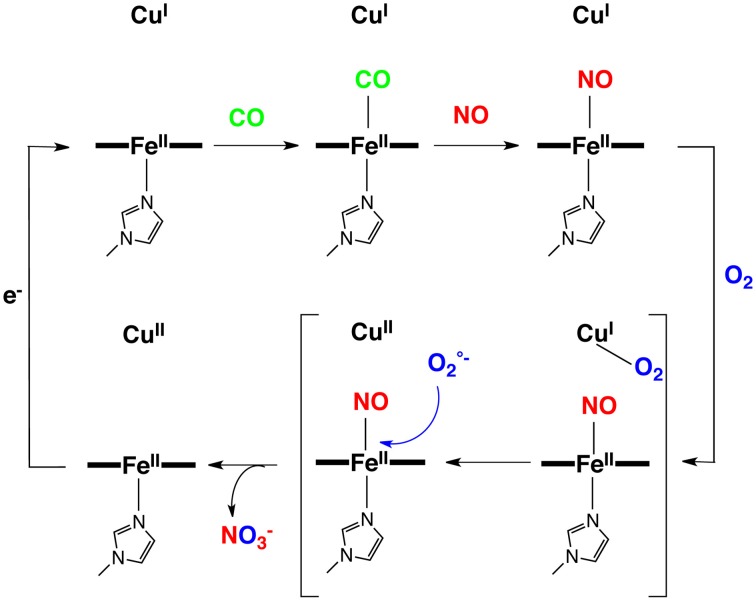
**Proposed reaction mechanism for the CN, CO, NO inhibitions**.

In summary NO, which is produced in the vicinity of CcO by mtNOS, may be a key actor in the defense of CcO against inhibition by both CO and CN^−^. On one hand, NO could replace these ligands to afford stable iron nitrosyl species, on the other hand NO has an extremely high binding affinity for reduced hemes. Hence, iron-nitrosyl species should be the end-product leading to CcO inhibition. But such a disaster does not occur because iron-nitrosyl species can be oxidized *in situ by* generation of superoxide formed through reaction of O_2_ with distal Cu(I) (Figure 9). Such a superoxide species (aca O_2_/Cu_B_) is also involved in the defense mechanism of various ligands against the oxidized enzyme, which may explain the increase of O${}_{2}^{-}$ concentration in CcO that is inhibited by ligand binding (Sipos et al., 2003). In the end O${}_{2}^{-}$/NO are a pair of endogenous ligands that protect CcO against external inhibitors. The former protects the reduced CcO active site and the later protects the oxidized CcO active site. This important lesson has been learned using functional model compounds.

#### Reactions with mitochondria

Studies on cyctochrome c oxidase itself, which was isolated using a well established procedure (Masters et al., 1965; Pearce et al., 2002, 2003), were carried out using the same a set of inhibitors: NO, CO, and CN^−^ (Pearce et al., 2008). These studies demonstate that the inhibitory effects of CO and CN^−^ are additive. On the other hand, NO appears to be antagonistic of the effect of CO and CN^−^ inhibitors. The activity of CcO in the presence of both NO and the inhibitors was ameliorated compared to the activity when NO was not present. Moreover, the displacement of CN^−^ in ferric hemoprotein by NO was found to be rate-limited by heme-reduction. Those results are consistent with the studies performed on our CcO model. This demonstrates that a synthetic model can be a useful tool to understand (or predict) inhibition of the enzyme.

### Hydrogen sulfide

#### Ligand

Hydrogen sulfide (H_2_S) is naturally produced in living organisms from L-cysteine by two cystathiones (gamma-lyase and beta-synthase) (Wang, 2002; Szabó, 2007), and is a gasotransmitter. In water H_2_S is known as a weak acid, hydrosulfuric acid or sulfhydric acid, giving the hydrosulfide ion (*pKa* = 6.9). It has a rotten egg odor and is also produced in volcanos (H_2_S is slightly heavier than air and air/H_2_S mixture may be explosive). Studies performed on organisms and isolated mitochondria showed that H_2_S is toxic at high concentrations (>600 ppm) (Khan et al., 1990; Dorman et al., 2002); whereas, at about 80 ppm H_2_S induces a state similar to hypothermia (mice body temperature 15°C) and a 90% decrease of metabolic rate (Blackstone et al., 2005; Blackstone and Roth, 2007; Lee, 2008; Volpato et al., 2008). Organisms are restored to normal physiological states once they inhalate fresh air (free of H_2_S); this phenomenon is reminiscent of hibernation (Heldmaier et al., 2004). Moreover, H_2_S is also involved in a series of physiological processes, such as blood pressure regulation, myocardial contractility, neurotransmission (Lowicka and Beltowski, 2007). In mitochondria, H_2_S at concentrations below the toxicity level (Beauchamp et al., 1984) was shown to be an hydrogen donor as well as a substrate for mitochondrial respiration. Moreover, H_2_S was demonstrated to be a reducing agent acting on the metal centers in CcO (Nicholls and Kim, 1982; Hill et al., 1984). These data suggest that, at least at low concentration, H_2_S is a non-competitive inhibitor of CcO (Petersen, 1977; Cooper and Brown, 2008).

#### Reaction with the CcO model

##### Characterized H_2_S-complexes

**Figure 10:**
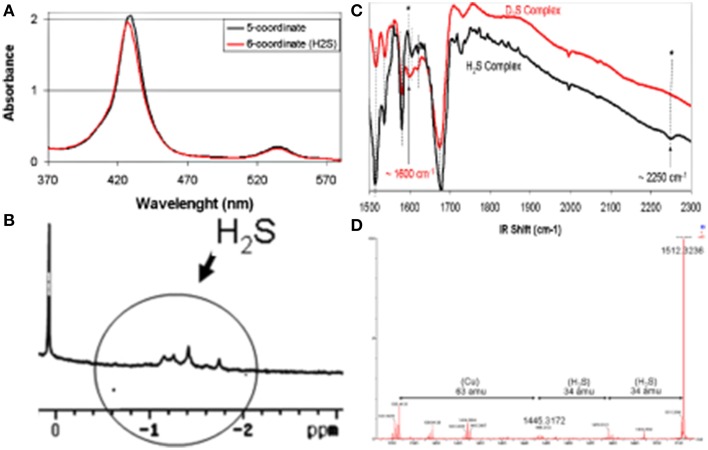
**Spectroscopic Characterizations of an FeCu H_2_S complex, (A) UV/Vis, (B) ^1^H-NMR, (C) IR, (D) HRMS**. Reproduced with permission from Collman et al. (2009d).

**Figure 11:**
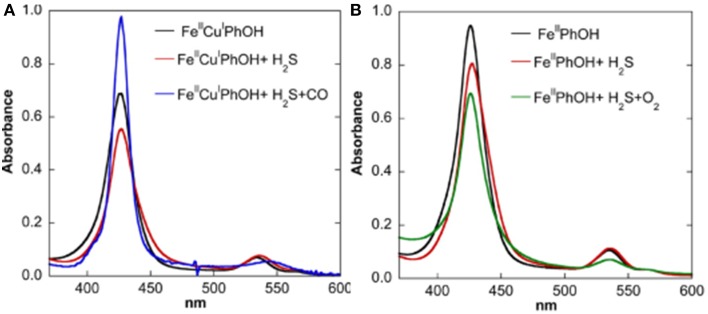
**Replacement of H_2_S: spectroscopic investigation**. UV/Vis Monitoring of the reactivity of the H_2_S complexes of Fe(II)Cu(I) (**A**, left) and Fe(II)-only (**B**, right) species [both containing the Tyr244 mimic (PhOH)] with CO, O_2_. Reproduced with permission from Collman et al. (2009d).

Reactions between H_2_S and metal complexes were first reported by Taube (Kuehn and Taube, 1976), Sellman (Sellman et al., 1991), and subsequently by our group (Collman et al., 2009d) and then others (Pavlik et al., 2010; Bennett et al., 2012; Miljkovic et al., 2013; Hartle et al., 2014; Ma et al., 2014). To examine the reactions and possible biomimetic reactivity of FeCu with this gaseous ligand, each individual metal site was first examined in a series of control reactions (Collman et al., 2009d). The iron-only species reacts with H_2_S to afford a complex, depicted as Fe-H_2_S, which was characterized by an array of spectroscopic tools (Figure 10). Upon addition of H_2_S to a solution of the anhydrous high-spin five-coordinate iron-only paramagnetic model, an H_2_S complex forms that contains two molecules of H_2_S in the distal pocket. The nature of the H_2_S-bound six-coordinate heme/Cu was supported by low-temperature ^1^H-NMR [diamagnetic features demonstrating a low-spin, six-coordinate system with upfield-shift of the H_2_S signals, where the protons are shielded in the anistropic cone, exhibiting a ring current (-1.1–1 ppm)]. HRMS (+34 amu) and MSMS Nanospray (bis-H_2_S/model adduct) with the D_2_S complex, UV/Vis (from five-coordinate (Soret: 430 nm) to six-coordinate (Soret: 427 nm), i.e., 10 nm shift in both the Soret and Q bands), infra-red (weak νS-H/D stretches at 2250/1600 cm^−1^ reminiscent of other stretches. Similarly the bimetallic FeCuPhOH model bearing a phenol moiety reacts with H_2_S affording an adduct depicted as FeCuPhOH-H_2_S with spectroscopic features comparable with that of the iron only case (UV/Vis, HRMS) (Figure 11). These derivatives are among the few H_2_S complexes reported so far having spectroscopic features comparable with those of other complexes (Kuehn and Taube, 1976; Anderson et al., 1977; Sellman et al., 1991; Muladige, 1994). Moreover, to examine a putative role of distal Cu in H_2_S binding, a modified model having Zn in the porphyrin and Cu in the distal site, i.e., ZnCu system did not show evidence of H_2_S binding. Altogether these findings establish that H_2_S binds to iron in the reduced active site.

##### Replacement of H_2_S

Studies in homogeneous solution showed that H_2_S is weakly bound as testified by binding constants of 12.5 and 10 μM, respectively. Subsequent competitive studies showed that this weak ligand is easily replaced by stronger ligands, such as CO or O_2_ (the resulting CO/O_2_ complexes were characterized by UV/Vis, ^1^H-NMR and Nanospray) (Collman et al., 2009d) (Figure 11). When the Fe-H_2_S complex was exposed to CO, another species formed Fe-H_2_S-CO, which has the spectroscopic features of an Fe-CO complex, such as UV/Vis absorption bands, its diamagnetic character allowed NMR characterization and IR stretches similar to those described previously (Collman et al., 2002). When Fe-H_2_S was exposed to O_2_, a complex forms that is designated as Fe-H_2_S-O_2_. The UV/Vis spectroscopic features of this intermediate correspond to Fe-O_2_ (Collman et al., 2003, 2007a, 2009a). These results indicate that H_2_S binding is weak and is easily replaced by ligands such as O_2_.

**Figure 12:**
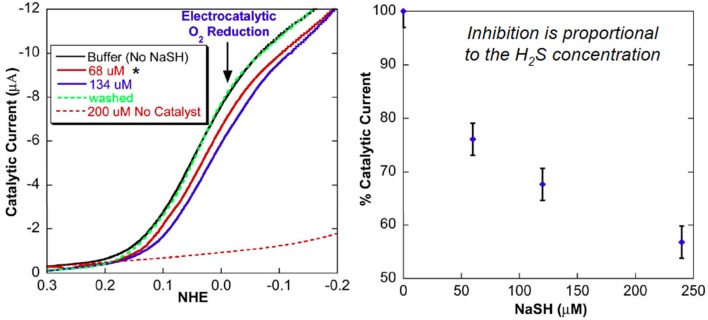
**Replacement of H_2_S: Electrocatalytic examinations**. Linear-sweep voltammogram of model 1 modified electrode. H_2_S reversibly inhibits the electrochemical catalytic reduction of O_2_. Reproduced with permission from Collman et al. (2009d).

Upon immobilization of our CcO model on a gold electrode, catalytic oxygen reduction was examined in an NaSH buffer solution, which served as a source of H_2_S (*pKa* = 7). The electrocatalytic current (representing O_2_ reduction) is significantly reduced. The electrocatalytic current gradually decreases as the H_2_S concentration was gradually increased (Figure 12). At 240 μM NaSH, the current is diminished by 60%. This inhibition is reversible: when H_2_S is removed by replacing the NaSH buffer with an air-saturated buffer, the catalytic O_2_ reduction at 0 mV vs. NHE is subsequently restored (Collman et al., 2009d). However, at low concentration of H_2_S, inhibition of oxygen reduction is not observed, presumably due to the low affinity of H_2_S for the Fe(II) hemes. This model study is indirectly related to experiments showing hibernation in mice.

##### H_2_S is also a reducing agent

**Figure 13:**
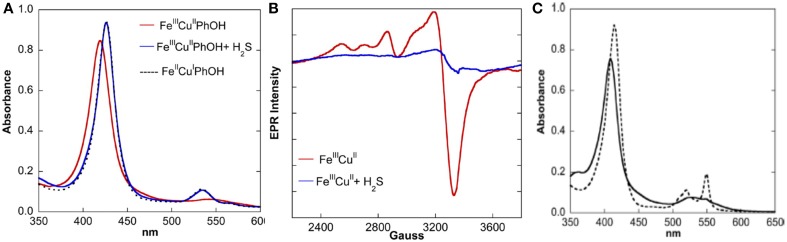
**H_2_S is a potent two-electron reducing agent: the reactivity of fully oxidized complex with H_2_S was monitored by UV/Vis (A, left) and EPR (B, middle) spectroscopies**. **(C)** (right): UV/Vis of oxidized Cyt. C (black) shows it could be reduced by H_2_S (dotted black). Reproduced with permission from Collman et al. (2009d).

Beside its ability to bind the fully reduced Fe(II)Cu(I) active site as a weak ligand, EPR and UV/Vis studies show that H_2_S can act as a powerful two-electron reducing agent (HS^−^ → S°+ H^+^ + 2e^−^; *E*° = 0.17 V at pH 7) (Collman et al., 2009d) (Figure 13). A solution of our fully oxidized Fe(III)Cu(II) complex (70 μM NaSH in water-acetonitrile), affords the Fe(II)Cu(I)-H_2_S species. These experiments were supported with characteristic UV/Vis absorption shifts in the Soret Band (410–428 nm) and Q bands [550 nm (broad) to 530 nm (sharp)] and also by EPR showing the disappearance of the 2200–3800 G signal (that corresponds to the overlap of low-spin ferric and cupric signals). Note that H_2_S (as a form of NaSH buffer) can also reduce Cyt. C, the e- reservoir of CcO.

### Non gaseous ligands

#### Ligands

A subsequent facet of our research focused on the reversible inhibition of both electrocatalytic oxygen reduction by CcO model and inhibition of mitochondrial respiration using non gaseous ligands (Collman et al., 2011; Barile et al., 2012). These abiological synthetic heterocycles are: substituted triazoles, tetrazoles, thiazoles, pyridine, etc… These were chosen as putative chelating ligands that would bind to both Fe and Cu in CcO's active site. Striking results were obtained with tetrazole (TZ).

#### Reactions with the models

**Figure 14:**
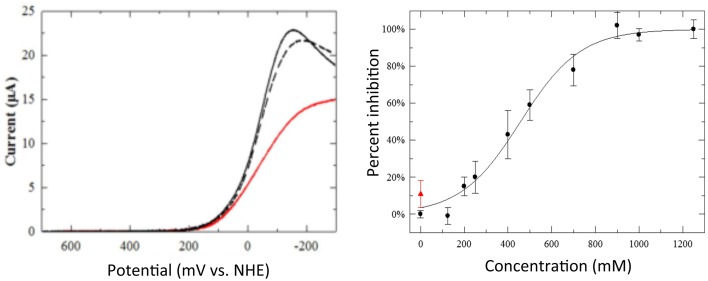
**Inhibition by heterocycles**. **Left**: linear sweep voltammograms showing the model electrocatalytic O_2_ reduction (solid black line), its inhibition by a 1-mM solution of tetrazole (TZ, red line), and the recovery of its catalyst after removing the solution of TZ (dotted black line). Thirty-four percent inhibition of peak current by 1-mM solutions of TZ. **Right**: percent inhibition of mitochondrial inhibition vs. concentration of TZ (black circles). Upon separating TZ from mitochondria by centrifugation and resuspending in buffer restores its respiration (red triangle). Reproduced with permission from Barile et al. (2012) and Collman et al. (2011).

Inhibition caused by small soluble molecules was studied by measuring the electrocatalytic O_2_ reduction of the model immobilized on slow-SAM-coated gold electrode. When no inhibitor is present, the catalytic O_2_ reduction proceeds normally (Figure 14, solid black line). Upon immersion in a solution of the inhibitor, inhibition of the model was detected as follows: the electrocatalytic O_2_ reduction current peaks occur at a greater overpotential and the current decreases (Figure 14, red line). However, the catalytic current is restored to almost its original value upon removing the inhibitor (Figure 14, dotted black line) (Collman et al., 2011; Barile et al., 2012).

#### Reaction with the mitochondria and with blood platelets

##### Mitochondria

The potency of TZ (and other heterocycles such as triazoles and thiazoles) to achieve mitochondrial inhibition was measured by its ability to reduce the rate of oxygen consumption (i.e., respiration) by using respiring mitochondria (Collman et al., 2011; Barile et al., 2012). The concentration of the compound that resulted in a 50% inhibition of respiration (IC50) was determined from a titration curve for each inhibitor. Upon inhibition, the mitochondrial suspension was then centrifuged affording a mitochondrial pellet that was subsequently washed and resuspended in fresh buffer: at this point the respiration rate was measured again. It was found that the respiration rate after such an inhibition/washing cycle was restored to near its original value with TZ (Figure 14, red triangle). These results indicated the compound's reversibility as an inhibitor of mitochondria. This reversibility of mitochondrial inhibition of such compounds of limited toxicity (Gross and Featherstone, 1946; O'Neal et al., 1978; Mizojiri et al., 1987; Aguilar et al., 2007) is very important compared to the irreversible and toxic inhibitors such as, azide, rotenone (Palmieri and Klingenberg, 1967; Degli Esposti, 1998; Alonso et al., 2003).

##### Platelets

**Figure 15:**
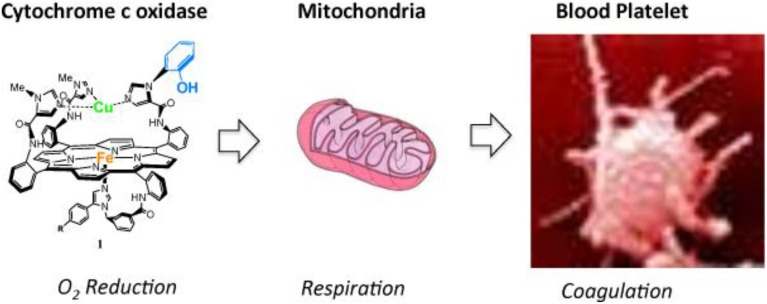
**Cytochrome c oxidase is found in mitochondria, mitochondria are found in blood platelets: a synthetic model of the Cytochrome c Oxidase active site may be a useful tool to address physiological processes in these systems**. Reproduced with permission from Decreau et al. (2010); BiologyCorner.com and Blausen.com staff. (“Blausen gallery 2014”. *Wikiversity J. Med*. Doi: 10.15347/wjm/2014.010. ISSN: 20018762), and 2015 Burlington Equine Veterinary Services, LLC.

The parallel correlation between the reversibilities of both the electrocatalytic O_2_ reduction by the model and the mitochondrial respiration illustrate the validity of using a functional model to predict behavior in living systems. Such a translational approach (connection to biological phenomena) was further illustrated by examining the inhibition of blood platelets with TZ (Figure 15). Blood platelets are known to undergo clumping and sticking processes (coagulation), and also to contain actively metabolizing mitochondria (Harmoning, 2009). Possible roles of platelet mitochondria in platelet function had been discussed, such as providing energy indirectely needed for platelet aggregation and secretion of procoagulant molecules, permeability of mitochondrial membranes linked to changes in coagulation activity (Salganicoff and Fukami, 1972; Hillman et al., 2005; Remenyi et al., 2005; Jobe et al., 2008). Inhibition of platelets results in the inhibition of clumping and sticking (anticoagulation). Hence, TZ was added to human whole blood, and subsequent platelet-activated clotting functions examined according to a known protocol (Pappas et al., 1994) led to interesting results: it was found that 50% inhibition occurs upon treatment with 1–100 mM of putative anticoagulants (Collman et al., 2011; Barile et al., 2012). The parallel correlation between mitochondrial inhibition and platelet inhibition suggests that the effect of the compounds on platelets function is mediated through mitochondria (Collman et al., 2011; Barile et al., 2012).

## Conclusion and perspectives

This study achieved two goals: (i) it developed a functional synthetic model of the CcO active site; (ii) the model is a useful tool to understand and predict CcO inhibition (and its offsets) by gases found in mitochondria (and by heterocycles).

The first stage of the study focused on the development (synthesis and O_2_-reduction studies) of the model containing key structural components of CcOs active site: the 4e^−^ reservoir mimicking heme *a3*, Cu_*B*_, and Tyr244. Subsequent single and steady-state turnover experiments showed that the model is functional. It passes through similar oxygenated intermediates (Oxy, and PM) meaning that this model also reduces O_2_ by four electrons. Upon immobilization on SAM-coated electrode (in SAM electron transfer is rate-limiting), the model achieves selective four-electron electrocatalytic reduction of O_2_ under physiological conditions (similar pH and potential, and rate-limiting electron transfer). Recent studies have showed that facets of this multi-component biomimetic tool could be improved. From the biomimetic “tool” perspective, i.e., the model on surface and subsequent electrocatalytic O_2_ reduction, a novel approach to carry out biomimetic studies is to employ diverse spectroscopic tools to identify reactive intermediates, such as coupling dynamic electrochemistry with surface-enhanced resonance Raman spectroscopy (SERRS) that allows the *in-situ* identification of O_2_-derived intermediates as they are formed on the electrode surface (Sengupta et al., 2013). Another important step in the elucidation of biomimetism is to mimic the protective hydrophobic environment around the catalytic site, because the CcO active site is buried in an hydrophobic bilayer. The initial electrocatalytic experiments carried out with the model immobilized on SAM-modified electrodes result in some hydrolytic autoxidation. Adding surfactants on top of the SAM film results in some decrease of the PROS that are formed. Moreover, a more systematic and sophisticated assembly has been prepared by depositing a phospholipid monolayer on top of the SAM, with the hydrophobic alkyl chains orientated inwards. The resulting catalyst ends up imbedded in a protective hybrid bilayer membrane (HBM) in which the lipid bilayer of the HBM removes all protic sources from the SAM-lipid interface. However, the incorporation of a proton carrier (i.e., decanoic acid) is required in order for the 4e^−^ O_2_ reduction to proceed (in such an HBM system, the O_2_ reduction current is lower and peaks at a more negative potential). As a result, the proton carrier accelerates the rate of proton transfer (from bulk solution to the HBM-imbeded catalyst) whereas the pH of the bulk solution influences the thermodynamics of the reaction (Hosseini et al., 2011).

In the second stage of the study, (i) this functional model was subsequently examined with spectroscopic and electrocatalytic measurements to understand the reversible inhibition of CcO by three gaseous ligands found in mitochondria: NO, CO, and H_2_S. A pair of protective endogenous ligands (O${}_{2}^{-}$/NO) have been identified to protect CcO against external inhibitors (such as CO, CN^−^): NO protects the reduced CcO active site and superoxide protects the oxidized CcO active site. Using our functional CcO model, we have demonstrated that H_2_S can reversibly inhibit catalytic oxygen reduction at the same concentration range that lowers the animal's temperature and slows respiration (hibernation). (ii) Other ligands were examined for the reaction with CcO, such as small heterocycles. A good correlation was found between the results from respiration studies on mitochondria and that from electrocatalytic studies on the model for a series of heterocycles inhibiting CcO. Interestingly, these electrocatalytic O_2_ reduction/respirometry tandem studies on mitochondria/synthetic model both demonstrated that tetrazole (TZ) was found to inhibit CcO reversibly. A similar correlation was established between these two reversible inhibitions (model, mitochondria) and the inhibition (deactivation) of platelets (mitochondria-rich key components of blood-coagulation). Hence, TZ appears to be an interesting class of anti-coagulants that inhibit platelet function, presumably by inhibiting mitochondrial respiration. Overall this set of experiments (on the model, mitochondria, and platelets, respectively) showed that a preliminary experiment carried out on the CcO model could predict the reversibility of the inhibition of respiration in mitochondria, and the deactivation in platelets. Such an approach is novel and may be useful in future studies examining the interaction of drugs with CcO. Future work will address the relative Kd for each ligand in the model compound and will be compared to that from mitochondria, which should give mitochondrial physiologists additional insight. This work is a rare example where connections between biological phenomena (such as respiration in mitochondria, and platelet clumping activity) and synthetic models have been achieved.

### Conflict of interest statement

The authors declare that the research was conducted in the absence of any commercial or financial relationships that could be construed as a potential conflict of interest.

## Abbreviations

AmN: Amyl Nitrite
ATP: adenosine triphosphate
CO: carbon monoxide
Cyt. C: Cytochrome c
CcO: Cytochrome c Oxidase
CN^−^: cyanide
Cu_A_: Copper-A
Cu_B_: distal Copper
EPR: Electron paramagnetic Resonance spectroscopy
5C: five-coordinate
6C: six-coordinate
Fe: iron
Fea3: heme a3
Fe-only, model with Fe in the porphyrin: and no metal in the distal position
FeCu, model with Fe in the porphyrin: and Cu in the tris-imidazole environment
HBM: hybrid bilayer membrane
HS: high-spin
H_2_S: hydrogen sulfide
His: Histidine
HRMS: high resolution mass spectrometry
IDA: inter-digitated array-electrodes
Kd: dissociation constant
Keq: equilibrium constant
kon: association rate constant
LS: low spin
Mb: myoglobin
mtNOS: mitochondrial Nitric Oxide Synthase
ν: wave number
^1^H-NMR: proton nuclear magnetic resonance
NO: nitric oxide
O_2_: dioxygen
O${}_{2}^{-}$: superoxide
PROS: partially reduced oxygen species
S: spin
SAM: self-assembled monolayer
RRDE: rotating ring-disk electrode
rR: resonance Raman
SERRS: surface-enhanced resonance raman scattering
Tyr: Tyrosine
TZ: (Tetrazole)
XAS: X-ray absorption spectroscopy.
